# Observation of the Effect of Singulair Combined With Ketotifen in the Treatment of Acute Exacerbation of Chronic Obstructive Pulmonary Disease With Airway Hyperresponsiveness and Its Influence on Th17/Treg

**DOI:** 10.3389/fsurg.2022.848724

**Published:** 2022-02-28

**Authors:** Haiou Wang, Gaojie Qu

**Affiliations:** The Third Affiliated Hospital, Department of Respiratory Medicine, Hengyang Medical School, University of South China, Hengyang, China

**Keywords:** acute exacerbation, chronic obstructive pulmonary disease, airway hyperresponsiveness, montelukast, ketotifen fumarate

## Abstract

**Objective:**

To investigate the effect of montelukast sodium (singulair) combined with ketotifen fumarate on the acute exacerbation of chronic obstructive pulmonary disease (AECOPD) with airway hyperresponsiveness (AHR) and its effect on helper T cells 17 (Th17)/regulator T cells (Treg).

**Methods:**

168 patients with AECOPD and AHR diagnosed in our hospital from February 2018 to December 2019 were selected, and divided into the observation group (*n* = 84) and the control group (*n* = 84). Both groups were given anti infection, bronchodilator, glucocorticoid, phosphodiesterase inhibitor, cough and expectorant. The observation group was additionally treated with singulair tablets and ketotifen tablets for 14 days. The curative effect were observed after treatment. The first second forced expiratory volume (FEV1), forced vital capacity (FVC) and FEV1 as percentage of predicted value (FEV1% pred), blood oxygen pressure (PaO_2_) and blood carbon dioxide pressure (PaCO_2_), high-sensitivity C-reactive protein (hs-CRP) and procalcitonin (PCT), Th17 and Treg levels were measured in both groups before and after treatment.

**Results:**

Compared with the control group, the total effective rate after treatment in the observation group was increased (94.05 vs. 75.00%, *P* < 0.05). Compared with before treatment, the FEV1, FVC and FEV1%pred levels of the two groups of patients after treatment were increased (*P* < 0.05). Compared with the control group, the FEV1, FVC and FEV1%pred levels of the observation group were increased after treatment (*P* < 0.05). Compared with before treatment, the PaCO2, hs-CRP and PCT levels of the two groups of patients were reduced after treatment, and PaO2 levels were increased (*P* < 0.05). Compared with the control group, the PaCO2, hs-CRP and PCT levels in the observation group were reduced after treatment, and the PaO2 level was increased (*P* < 0.05). Compared with before treatment, Th17 and Th17/Treg levels of the two groups of patients were reduced after treatment, and Treg levels were increased (*P* < 0.05). Compared with the control group, the Th17 and Th17/Treg levels of the observation group were reduced after treatment, and the Treg levels was increased (*P* < 0.05).

**Conclusion:**

Singulair combined with ketotifen in the treatment of patients with AECOPD combined with AHR can significantly improve the efficacy, improve lung function, reduce inflammatory response, and improve the balance of Th17/Treg, effectively controlling the disease.

## Introduction

Chronic obstructive pulmonary disease (COPD) is a common pulmonary disease with the characteristics of airflow limitation, which develops irreversibly and progressive (1, 2). COPD is more common in middle-aged people, especially in people with a long history of smoking or long-term exposure to other harmful gases. Its clinical symptoms include chronic cough, expectoration, shortness of breath or dyspnea. In the early stage, it only appears during severe exercise, but with the aggravation of the disease, it often appears in daily life (3, 4). Acute exacerbation of COPD (AECOPD) is a severe stage in the course of COPD, in which the exacerbation of respiratory symptoms exceeds the daily level, which can be caused by bacterial infection, changes in environmental factors, and other reasons (5, 6). Airway hyper responsiveness (AHR) refers to the strong contraction response of the airway after being exposed to stimulation factors, which is often accompanied by AECOPD. In recent years, due to the aggravation of environmental pollution and the aging process of population in China, the incidence of AECOPD combined with AHR is gradually increasing (7, 8). It has become extremely important to select the appropriate and effective treatment for AECOPD combined with AHR. Montelukast sodium (singulair) is an oral leukotriene (LT) receptor antagonist that inhibits the binding of LT to its receptors in the airway, thereby rendering LT ineffective in improving airway inflammation (9, 10). Ketotifen fumarate tablets, as an allergic mediator release inhibitor for sensitized active cell mast cells or basophils, can reduce the release of allergic active mediator and have good anti-allergic effect (11, 12). T helper cell 17 (Th17) is a kind of pro-inflammatory cell that plays an important role in the body's own defense (13). T regulatory cells (Treg) are generated by thymus and can inhibit the proliferation of autoreactive T cells in the body and play a negative role in immune regulation (14). The purpose of this study was to investigate the efficacy of singulair in combination with ketotifen in the treatment of AECOPD with AHR and its effect on Th17/Treg.

## Materials and Methods

### Patients

A total of 168 patients with AECOPD and AHR diagnosed in our hospital from February 2018 to December 2019 were selected. Inclusion criteria: All patients met the diagnostic guidelines for AECOPD (15); no recent oral glucocorticoid or ICS therapy; no history of bronchial asthma. Determination of AHR by bronchial provocation test (16): First, the forced vital capacity curve of the subject was determined with the pulmonary function meter, and the first second forced expiratory volume (FEV1) of the three curve was taken as the basic value. The provocation test could be performed for patients with FEV1 > 70%. The lung function was measured after the subjects were inhaled with normal saline. If the FEV1 decreased by more than 10% after the normal saline was inhaled, the airway reactivity would be high and the test would not be appropriate. If FEV1 decreased by no more than 10%, subjects were given histamine at an increasing dose of 5% beginning with the lower dose, and pulmonary ventilation was measured after inhalation until FEV1 decreased by more than 20% from baseline or symptoms appeared, or the maximum dose of histamine was inhaled. After histamine stimulation, albuterol was routinely inhaled, and the positive person would be AHR. Exclusion criteria: patients combined with severe cardiac insufficiency and arrhythmia; patients with severe cerebrovascular diseases; patients combined with severe hyperthyroidism, patients with pulmonary infection; patients who smoke. A total of 168 AECOPD patients complicated with AHR were randomly divided into the observation group (*n* = 84) and the control group (*n* = 84). There was no significant difference in general data between the two groups (*P* < 0.05).

### Research Methods

The control group received sensitive antibiotics to fight infection according to the sputum culture drug sensitivity test, aminophylline, salbutamol, budesonide solution to relieve asthma, ambroxol expectorant, oxygen inhalation, etc., and systemic use of glucocorticoid (methylprednisolone), which was discontinued after infection control. On this basis, the observation group was additionally treated with singulair tablets (Hangzhou MSD Pharmaceutical Co., LTD.) 10 mg orally, once a day, every night before bedtime. Ketotifen tablets (Jiangsu Pengrier Pharmaceutical Co., LTD.) were orally taken at 1 mg, twice a day. The treatment course was 14 days. Evaluation criteria for therapeutic effects: Excellent: The symptoms such as cough and wheeze disappeared, and the dry and wet rales in the lung disappeared. Effective: cough and wheeze are reduced, and pulmonary dry and wet rales are reduced; Ineffective: cough and wheeze were not improved obviously, and pulmonary dry and wet rales recurred. Pulmonary function tests were carried out, respectively, and the PowerCube spirometer of German Kangxun Company was used to check the FEV1, FVC, and FEV1%pred before and after treatment in the two groups. Each inspection is determined by a professional technician. The ABL90 blood gas analyzer provided by Radiometer Medical Equipment (Shanghai) Co., Ltd. was used to analyze the blood gas of the patient's blood to determine the PaO_2_ and PaCO_2_. The venous blood of the two groups of patients was drawn, and the hs-CRP and PCT were determined by ELISA. The percentage contents of Th17 and Treg cells in the venous blood of patients were detected by flow cytometry.

### Statistical Methods

All data were processed with SPSS 22.0 statistical software. The enumeration data were examined by X^2^ test and expressed by [*n* (%)], the measurement data were examined by *t*-test and expressed by ($\overset{\bar{}}{x}$ ± s). The difference is statistically significant when *P* < 0.05.

## Results

### Comparison of Clinical Effects Between the Two Groups

**Figure 1:**
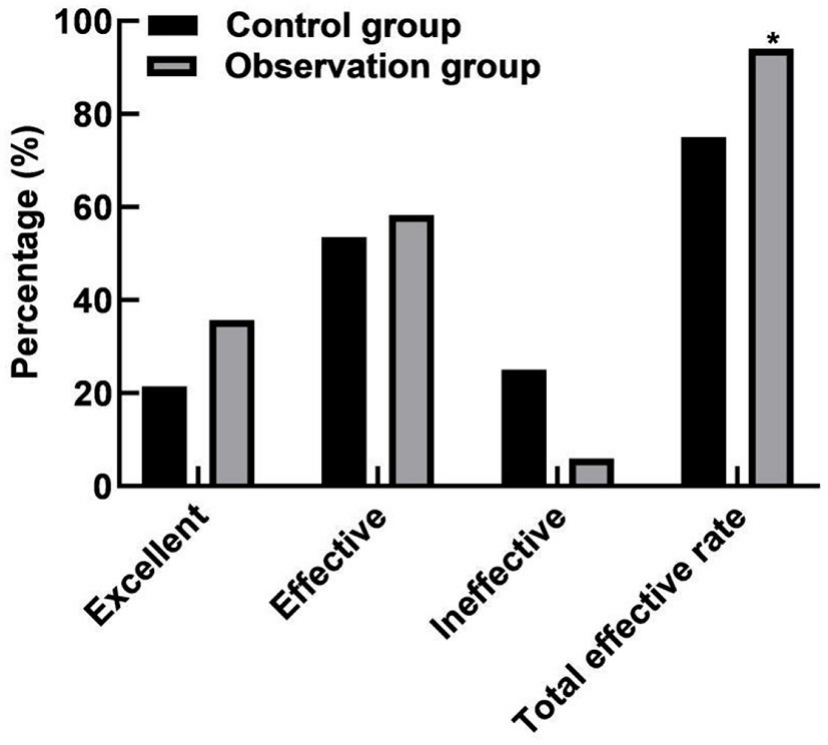
Comparison of clinical effects between the two groups. Compared with the control group, *P < 0.05.

Compared with the control group, the total effective rate after treatment in the observation group was increased (94.05 vs. 75.00%, *P* < 0.05). As shown in Figure 1.

### Comparison of Lung Function Between the Two Groups

**Figure 2:**
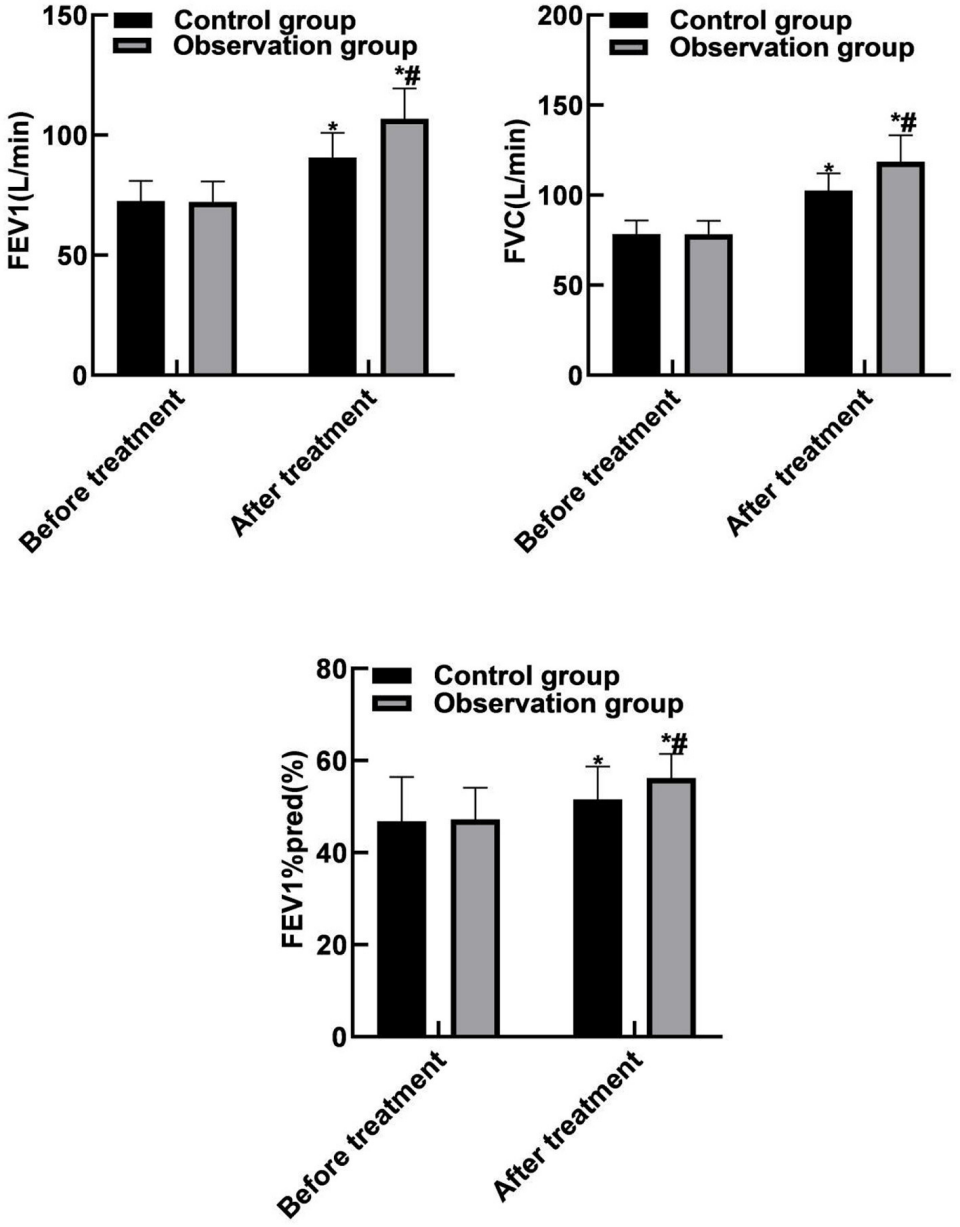
Comparison of lung function between the two groups. Compared with before treatment, *P < 0.05. Compared with the control group, ^#^*P* < 0.05.

Compared with before treatment, the FEV1, FVC, and FEV1%pred levels of the two groups of patients were increased after treatment (*P* < 0.05). Compared with the control group, the FEV1, FVC, and FEV1%pred levels of the observation group increased after treatment (*P* < 0.05). As shown in Figure 2.

### Comparison of Blood Gas Indicators and Inflammation Indicators Between the Two Groups

**Figure 3:**
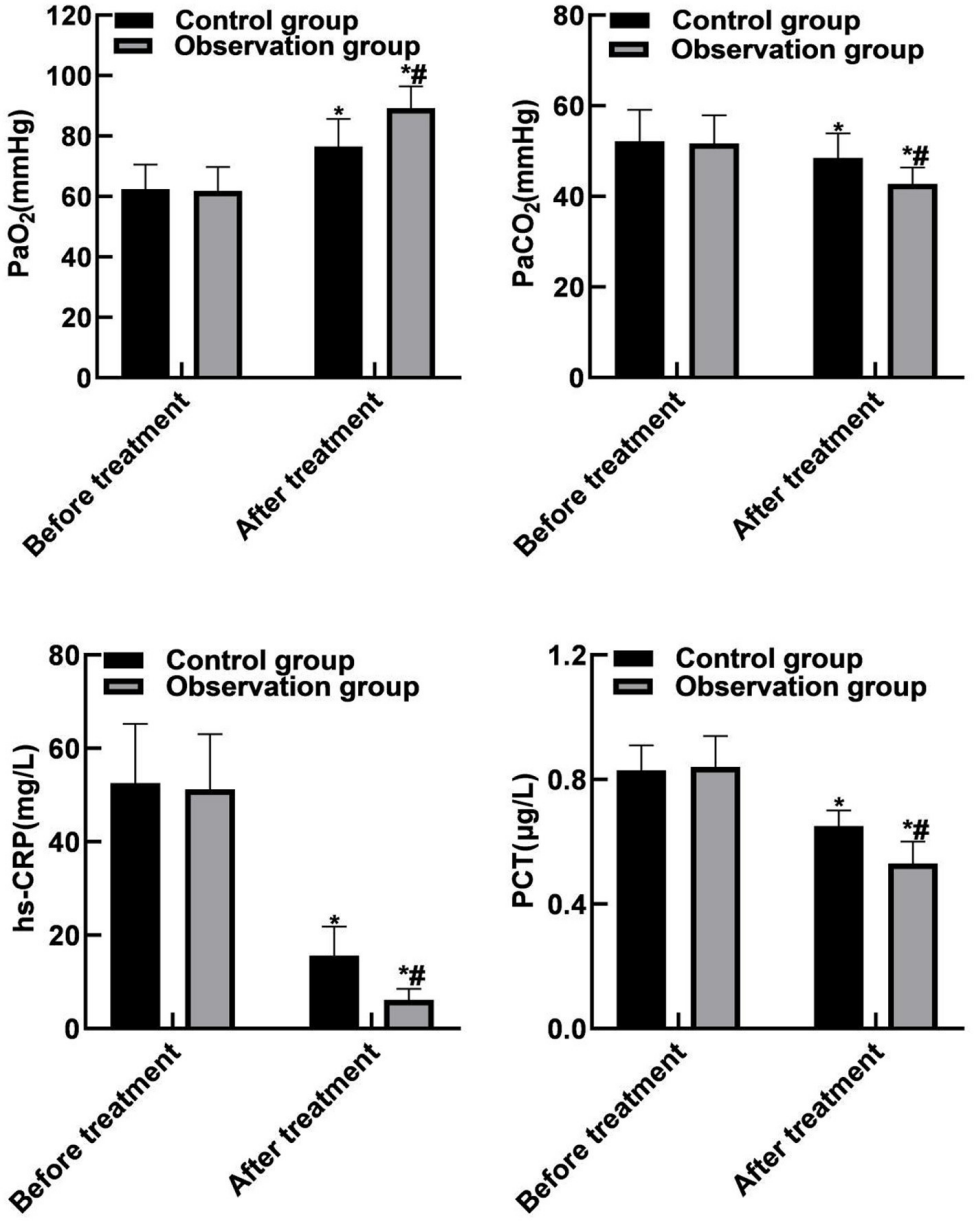
Comparison of blood gas indicators and inflammation indicators between the two groups. Compared with before treatment, *P < 0.05. Compared with the control group, ^#^*P* < 0.05.

Compared with before treatment, the PaCO_2_, hs-CRP and PCT levels of the two groups of patients were reduced after treatment, and PaO_2_ levels were increased (P <0.05). Compared with the control group, the PaCO_2_, hs-CRP and PCT levels in the observation group were reduced after treatment, and the PaO_2_ level was increased (*P* < 0.05). As shown in Figure 3.

### Comparison of Th17 and Treg Between the Two Groups

**Figure 4:**
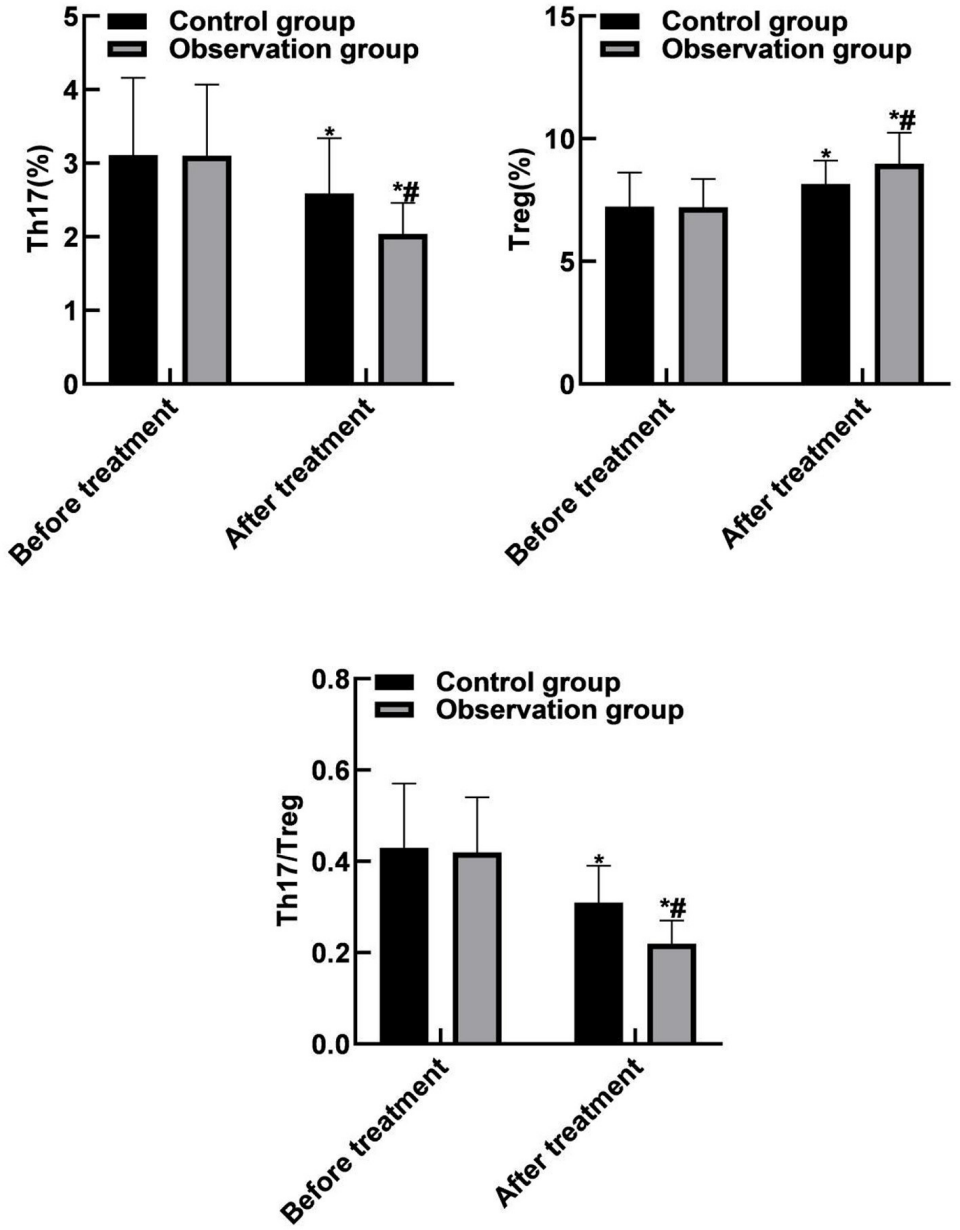
Comparison of Th17 and Treg between the two groups. Compared with before treatment, *P < 0.05. Compared with the control group, ^#^*P* < 0.05.

Compared with before treatment, Th17 and Th17/Treg levels of the two groups of patients were reduced after treatment, and Treg levels were increased (*P* < 0.05). Compared with the control group, the Th17 and Th17/Treg levels of the observation group were reduced after treatment, and the Treg levels was increased (*P* < 0.05). As shown in Figure 4.

## Discussion

COPD is a common chronic lung disease, and its pathogenesis is abnormal inflammatory reaction caused by lung infection due to long-term smoking or external environmental factors such as respiratory tract infection, which causes excessive mucus secretion to narrow the airway and aggravate the obstruction (17–19). AECOPD is a serious stage in the course of COPD, in which the inflammatory response is aggravated, resulting in the increase of eosinophils and basophils in the body, and the release of more neutrophil chemokines and proteolytic enzymes to participate in the inflammatory response, and the persistent inflammatory response may even lead to the death of the patient (20, 21). AHR is often associated with COPD, and it is caused by narrowing and obstruction of the airway in patients with COPD that the mucosal epithelium of the airway is damaged, resulting in the enhanced response of the airway to non-specific stimuli, further triggering inflammation (22, 23). Therefore, selecting treatment that is effective in alleviating AHR is one of the main strategies for preventing and treating the progression of COPD.

The results of this study showed that compared with the control group, the total effective rate of the observation group was higher after treatment. The reason for this was analyzed as LT is an inflammatory response substance different from glucocorticoid-sensitive mediators, which has a high biological activity and always exists in the occurrence and development of COPD. Singulair is a specific cysteine LT receptor antagonist, which can effectively inhibit the pro-maturation effect of peptide growth factor on eosinophilic and basophilic stem cells, and reduce the eosinophils in the airway and surrounding blood, thereby reducing airway inflammation, relaxing smooth muscle, and reducing pulmonary hypertension (24, 25). Ketotifen is an allergy-inducing medium release inhibitor of mast cells or basophils, which can protect the membrane of mast cells or basophils, reduce the membrane allosterism under the attack of allergens, and prevent the release of allergic reaction medium. At the same time, it has strong antihistamine H1 receptor antagonism, which can effectively inhibit the release of histamine by bronchial submucosal mast cells to avoid the occurrence of respiratory tract non-specific inflammation, and thus reduce AHR (26, 27).

The results of this study showed that compared with before treatment, the levels of FEV1, FVC, and FEV1%pred in the two groups were increased after treatment. Compared with the control group, the FEV1, FVC, and FEV1%pred levels of the observation group increased after treatment. These results indicated that the combination therapy of singulair and ketotifen could effectively improve the lung function of patients. LT is the medium that causes increased mucus secretion. Singulair can prevent the adhesion of white blood cells and endothelial cells, reduce the production of inflammatory mediators, various proteases and oxygen free radicals, thereby inhibiting airway inflammation, reducing mucus secretion, reducing airway hyperresponsiveness and relieving airway spasm. The results of this study showed that compared with before treatment, the PaCO_2_, hs-CRP and PCT levels of the two groups of patients were reduced after treatment, and PaO_2_ levels were increased. Compared with the control group, the PaCO_2_, hs-CRP and PCT levels of the observation group were reduced after treatment, and the PaO_2_ level was increased. The reason was analyzed as follows: Singulair could effectively inhibit the binding of LT to its receptor in airway and make it unable to play a role, thus reducing the permeability of capillaries, reducing mucus secretion, relieving airway inflammation and improving the ventilatory function and inflammatory reaction of patients.

Th17 cells are a subpopulation of Th cells and are named after their ability to produce IL-17. As a pre-inflammatory factor, IL-17 is involved in many inflammatory responses and in a variety of autoimmune diseases. Activated Th17 cells can secrete IL-17, which combines with inflammatory cell surface receptors to induce the release of a large number of inflammatory factors that act on the airway mucus glands to produce a large amount of mucus, increase airway responsiveness, and further promote airway remodeling (28, 29). Treg cells, as important regulatory cells in the body, can negatively regulate T cells and reduce body damage caused by over-immunity of antigen and antibody. Treg cells are related to the immune function of patients, and can indirectly reflect the degree of inflammation in the lungs and airways. The increase of Treg cells causes immune imbalance, and then inhibits the increase of Th17 cell activity, weakening the inflammatory response in the lungs, which may induce lung cancer. However, the mechanism needs further research and discovery. Similar to the balance between T helper 1 cell (Th1) and T helper 2 cell (Th2), there is also an important balance between Th17 and Treg cells. The imbalance will lead to local or systemic abnormal immune responses, such as autoimmune diseases, tumors, persistent infections and other diseases. At present, more and more pulmonary diseases have been found to be related to immune disorders, especially the role of Th17/Treg cells in the pathogenesis of pulmonary diseases has received increasing attention (30, 31). The results of this study showed that compared with before treatment, Th17 and Th17/Treg levels of the two groups of patients were reduced after treatment, and Treg levels were increased. Compared with the control group, the Th17 and Th17/Treg levels of the observation group were reduced after treatment, and the Treg levels was increased. These results indicated that the combination therapy of singulair and ketotifen could improve the Th17/Treg balance and maintain the immune balance of the body, thereby effectively improving the disease condition.

In summary, singulair combined with ketotifen in the treatment of patients with AECOPD combined with AHR can significantly improve the curative effect, improve the lung function of patients, reduce the inflammatory response, and improve the Th17/Treg balance, and effectively control the disease.

## Data Availability Statement

The original contributions presented in the study are included in the article/supplementary material, further inquiries can be directed to the corresponding author/s.

## Ethics Statement

The studies involving human participants were reviewed and approved by the Ethics Committee of the Third Affiliated Hospital of South China University. The patients/participants provided their written informed consent to participate in this study.

## Author Contributions

Both authors have made equal contributions and participated in the design of the study, the search for literature, the analysis of the data, and the writing and revision of the paper. Both authors contributed to the article and approved the submitted version.

## Conflict of Interest

The authors declare that the research was conducted in the absence of any commercial or financial relationships that could be construed as a potential conflict of interest.

## Publisher's Note

All claims expressed in this article are solely those of the authors and do not necessarily represent those of their affiliated organizations, or those of the publisher, the editors and the reviewers. Any product that may be evaluated in this article, or claim that may be made by its manufacturer, is not guaranteed or endorsed by the publisher.
